# Marizomib: A novel therapeutic approach for the treatment of central nervous system myeloma

**DOI:** 10.1002/jha2.72

**Published:** 2020-08-12

**Authors:** Despina Bazou, Giao Le, Aoife Boyle, Agnieszka Blum, Peter O'Gorman

**Affiliations:** ^1^ Department of Haematology Mater Misericordiae University Hospital Dublin 7 Dublin Ireland

Multiple myeloma is a malignant plasma cell disorder characterized by abnormal proliferation of clonal plasma cells in the bone marrow microenvironment. Involvement of the central nervous system is a rare extramedullary manifestation of multiple myeloma, occurring in approximately 1% of patients and confers a poor prognosis with a median overall survival of 2‐4 months [1, 2]. CNS myeloma is defined by the presence of malignant plasma cells in the cerebrospinal fluid (CSF) and/or leptomeningeal, dura mater, or intraparenchymatous involvement [3]. While novel therapies such as immunomodulatory agents, proteasome inhibitors, and monoclonal antibodies have significantly improved the overall survival of MM in recent years, their role in CNS myeloma has not been well defined. Bortezomib, the first‐generation proteasome inhibitor, has not shown evidence that it can cross the blood‐brain barrier (BBB). Immunomodulators such as thalidomide, lenalidomide, and pomalidomide are able to penetrate the BBB, as evidenced by their central neurotoxic effects such as somnolence and sedation particularly with thalidomide. Drug levels of thalidomide and lenalidomide were measurable in CSF of nonhuman primates after oral administration [4]. Preclinical studies of murine models have shown promising potential of pomalidomide in CNS lymphoma [5], however, there is limited data on the effectiveness of immunomodulators in CNS myeloma. Here, we describe the novel therapeutic use of marizomib, an irreversible proteasome inhibitor that crosses the BBB and has demonstrated a significant survival benefit in a patient with CNS myeloma.

A 35‐year‐old Caucasian female with no significant past medical history presented to her general practitioner in September 2012 with a 2‐month history of epistaxis, abdominal pain, menorrhagia, diarrhea, and malaise. Initial laboratory results showed anemia, thrombocytopenia, hypercalcemia, and mild renal impairment. Subsequent investigations by hematology service showed an IgG kappa M‐protein of 72 g/L with the presence of immunoparesis on serum protein electrophoresis (SPEP) and immunofixation, kappa light chain of 3580 g/L, lambda light chain of <0.1 mg/L, albumin 33 g/l, and β2 microglobulin 1.8 mg/L. She had 80% CD138 positive plasma cells infiltrate in her bone marrow, with a complex hypodiploid clone (di‐centric chromosome involving chromosome X and 8, additional material on chromosomes 2, 5, 11, 13, and aberrations involving chromosomes 6, 9, 12, 13). Whole body MRI showed bone marrow packing in the axial and appendicular skeleton, while PET‐CT showed focal concentration of FDG avidity in the right sacral bone. She was diagnosed with ISS stage 2 symptomatic myeloma.

In October 2012, she began induction therapy with six cycles of lenalidomide, bortezomib, and dexamethasone (RVD), followed by high‐dose melphalan and autologous stem cell transplantation (ASCT) in June 2013, achieving complete response. Subsequently, she was commenced on high‐risk maintenance therapy with lenalidomide and bortezomib.

**FIGURE 1:**
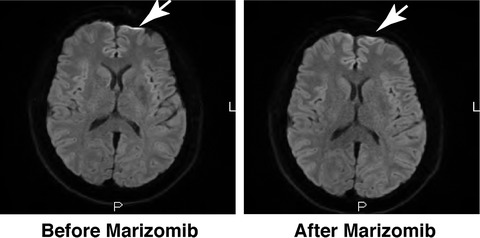
MRI brain showed CNS infiltration with leptomeningeal enhancement (before Marizomib, arrow) and resolution of the leptomeningeal enhancement post marizomib administration

In January 2016, she had clinical and biochemical relapse, and was treated with pomalidomide, bortezomib, and low‐dose dexamethasone (PVD). Clonal evolution was evident in her plasma cells showing del(17p), t(4;14), 1q gain, and, monosomy 13, confirming aggressive disease at relapse. Despite a three‐drug combination treatment, her disease progressed 4 months later, and she was salvaged with daratumumab. In September 2016, she presented with new‐onset neurological symptoms of headache and dizziness. Neurological examination was remarkable for bilateral papilledema. MRI brain showed CNS infiltration with leptomeningeal enhancement and MRI whole spine revealed an abnormal signal in the conus medullaris (Figure 1). Cytologic examination of her CSF was positive for malignant plasma cells, confirming CNS myeloma.

Following confirmation of myelomatous CNS involvement, the patient initially received pulsed methylprednisolone 1 mg/kg IV for 3 days followed by radiotherapy, which resulted in a transient symptomatic improvement. Subsequently, she was commenced on intrathecal chemotherapy regimen (methotrexate, cytarabine, and hydrocortisone). However, her neurological symptoms returned, associating with a persistent presence of malignant plasma cells in her CSF. Pomalidomide was restarted along with daratumumab for synergistic effect to control her systemic progression.

**FIGURE 2:**
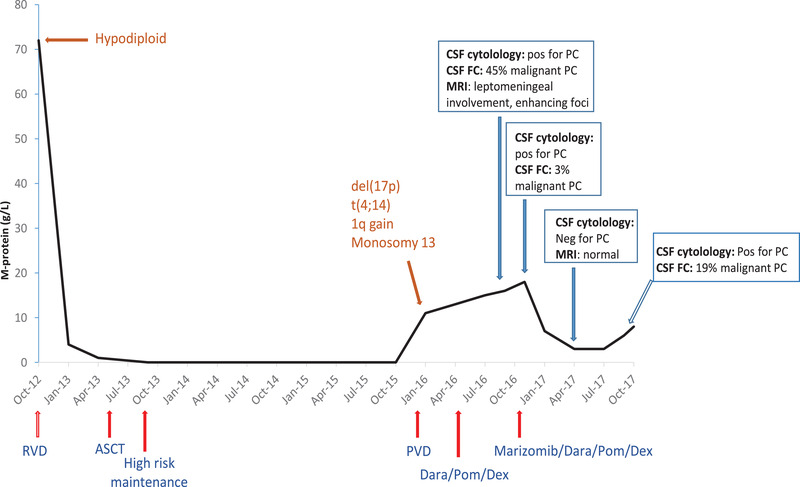
Graphical depiction of serum M‐protein measurements throughout the patient's disease course. Treatment regimens are shown below the time line. Cytogenetics at diagnosis and at relapse are highlighted. Evidence of CNS involvement starting in September 2016 depends on clinical symptoms, CSF cytology, CSF flow cytometry (FC), and MRI

In November 2016, she was commenced on marizomib 0.7 mg/m^2^ on days 1, 8, 15, and dexamethasone 10 mg on days 1, 2, 8, 9, 15, and 16 on a compassionate use basis. Salvage therapy with marizomib was well tolerated with no adverse events and resulted in a sustained clinical and radiological response and a reduction in CNS malignant plasmacytosis. After five cycles of marizomib, the patient's neurological symptoms of headaches and dizziness resolved. By cycle 7, her MRI brain demonstrated radiological improvement with no abnormal findings and complete resolution of leptomeningeal enhancement. CSF cytology was negative for malignant plasma cells. She also achieved partial response with a plateauing M‐protein at 3 g/L. However, 10 months after starting marizomib, her disease rapidly progressed systemically with the recurrence of myelomatous infiltration in her CSF, and she died in December 2017 (Figure 2).

In this letter, we showed that marizomib, a novel third generation proteasome inhibitor with activity and specificity that is distinct from other proteasome inhibitors, has the potential to fulfil this unmet clinical need in patients with CNS myeloma. Marizomib has the unique advantage of being the first PI that can irreversibly inhibit all three proteasome enzymatic subunits and can penetrate the blood brain barrier. Several studies have shown that marizomib can be retained in the CNS and substantially inhibits proteasome activity in the brain [6, 7]. A preclinical study reported that radiolabeled marizomib showed 30% CNS biodistribution compared with blood levels in rats, eliciting a significant antitumor effect in rodent models of malignant glioma [6].

Here, the administration of marizomib resulted in a rapid and sustained clinical and radiological improvement with resolution of neurological symptoms and leptomeningeal enhancement, respectively. Furthermore, it led to >90% reduction in CSF plasmacytosis with a survival benefit of 1 year, which is three times that of the reported median survival [8, 9]. In terms of safety profile, marizomib was well tolerated and did not exhibit any adverse events such as hematological toxicity or peripheral neuropathy that are frequently observed with bortezomib and carfilzomib. In conclusion, this case provides further evidence for marizomib as a potential leading therapeutic agent in CNS myeloma.
